# Avoidable mortality from giving tranexamic acid to bleeding trauma patients: an estimation based on WHO mortality data, a systematic literature review and data from the CRASH-2 trial

**DOI:** 10.1186/1471-227X-12-3

**Published:** 2012-03-01

**Authors:** Katharine Ker, Junko Kiriya, Pablo Perel, Phil Edwards, Haleema Shakur, Ian Roberts

**Affiliations:** 1 Clinical Trials Unit, Faculty of Epidemiology & Population Health, London School of Hygiene & Tropical Medicine, Keppel Street, London WC1E 7HT, UK

## Abstract

**Background:**

The CRASH-2 trial showed that early administration of tranexamic acid (TXA) safely reduces mortality in bleeding in trauma patients. Based on data from the CRASH-2 trial, global mortality data and a systematic literature review, we estimated the number of premature deaths that might be averted every year worldwide through the use of TXA.

**Methods:**

We used CRASH-2 trial data to examine the effect of TXA on death due to bleeding by geographical region. We used WHO mortality data (2008) and data from a systematic review of the literature to estimate the annual number of in-hospital trauma deaths due to bleeding. We then used the relative risk estimates from the CRASH-2 trial to estimate the number of premature deaths that could be averted if all hospitalised bleeding trauma patients received TXA within one hour of injury, and within three hours of injury. Sensitivity analyses were used to explore the effect of uncertainty in the parameter estimates and the assumptions made in the model.

**Results:**

There is no evidence that the effect of TXA on death due to bleeding varies by geographical region (heterogeneity *p *= 0.70). Based on WHO data and our systematic literature review, we estimate that each year worldwide there are approximately 400,000 in-hospital trauma deaths due to bleeding. If patients received TXA within one hour of injury then approximately 128,000 (uncertainty range [UR] ≈ 72,000 to 172,000) deaths might be averted. If patients received TXA within three hours of injury then approximately 112,000 (UR ≈ 68,000 to 148,000) deaths might be averted. Country specific estimates show that the largest numbers of deaths averted would be in India and China.

**Conclusions:**

The use of TXA in the treatment of traumatic bleeding has the potential to prevent many premature deaths every year. A large proportion of the potential health gains are in low and middle income countries.

## Background

Trauma is a leading cause of death and disability. Each year, worldwide, an estimated 5.8 million people die as a result of trauma [1], many after reaching hospital. Among trauma patients who survive to reach hospital, bleeding is a common cause of death, accounting for around 40% of in-hospital trauma deaths [2].

The CRASH-2 trial was an international randomised controlled trial of the early administration of tranexamic acid (TXA) to bleeding trauma patients. The trial recruited 20,211 patients from 274 hospitals in 40 countries. The results show that TXA reduces mortality in trauma patients with or at risk of bleeding, with no apparent increase in side effects [3]. If given within three hours of injury, TXA reduces the risk of death due to bleeding by about a third [4]. TXA administration has been shown to be highly cost-effective in high, middle or low income countries [5]. On the basis of the results of the CRASH-2 trial, TXA has been included on the WHO Essential Medicines List [6].

Since publication of the trial results, TXA has been included into trauma care guidelines in many high income countries. In March 2010, the British Army incorporated TXA into combat care treatment protocols [7] and in July 2011 the UK NHS ambulance service agreed that TXA would be given to all adults and teenagers who suffer major injury in the UK. In 2011, the US Army reviewed the evidence from the CRASH-2 trial and included TXA into its trauma treatment protocols. However, bearing in mind that 90% of trauma deaths are in low and middle income countries [8], the potential of TXA to reduce premature mortality is likely to be much greater in these settings. An estimation of the number of deaths that could be averted through the use of TXA for in traumatic haemorrhage would allow better targeting of dissemination and implementation activities. In this study we used data from the CRASH-2 trial, WHO mortality database and a systematic review of the recent literature, to estimate the potential number of deaths that could be averted through the early administration of TXA to bleeding trauma patients.

## Methods

### Estimation of effect of TXA on death due to bleeding by geographical region

We used individual patient data from the CRASH-2 trial to assess the extent to which the effect of TXA on death due to bleeding varied according to geographical region. Hospitals participating in the CRASH-2 trial were grouped into four geographical regions: (1) Africa, (2) Asia, (3) Europe, Australia, North America, and (4) Central & South America. Heterogeneity in treatment effect by geographical region was assessed by a χ^2 ^test. We pre-specified that unless there was strong evidence against the null hypothesis of homogeneity of effects (i.e. *p *< 0.001), the overall risk ratio (RR) would be considered to be the most reliable guide to the approximate RRs in all regions.

### Estimation of number of in-hospital trauma deaths due to bleeding per year

The number of in-hospital trauma deaths that are due to bleeding and thus potentially avoidable through the early administration of TXA was estimated in three steps.

First, we obtained estimates of the number of trauma deaths (N_T_) by country. Since the risk of death due to bleeding may vary according to type of injury (i.e. blunt or penetrating) [9], we classified deaths as being a result of blunt trauma (N_BT_) or penetrating trauma (N_PT_). Second, we obtained data on the proportion of trauma deaths that occur in hospital (P_H_). The numbers of in-hospital blunt trauma deaths (N_H, BT_) and in-hospital penetrating trauma deaths (N_H, PT_) were then estimated according to the following equations:

$$\begin{array}{c} {\text{N}}_{\text{H,BT}}={\text{N}}_{\text{BT}}\times {\text{P}}_{\text{H}}\\ {\text{N}}_{\text{H,PT}}={\text{N}}_{\text{PT}}\times {\text{P}}_{\text{H}} \end{array}$$

Third, we obtained data on proportion of in-hospital blunt trauma deaths caused by bleeding (P_H, BT, BL_) and the proportion of in-hospital penetrating trauma deaths caused by bleeding (P_H, PT, BL_). Using these estimates we derived the number of in-hospital trauma deaths caused by bleeding (N_H, T, BL_) as follows;

$${\text{N}}_{\text{H,T,BL}}=\left({\text{N}}_{\text{H,BT}}\times {\text{P}}_{\text{H,BT,BL}}\right)+\left({\text{N}}_{\text{H,PT}}\times {\text{P}}_{\text{H,PT,BL}}\right)$$

The number of premature deaths potentially averted by TXA was then estimated by applying the relative risk reduction from the CRASH-2 trial to the number of in-hospital deaths due to bleeding as follows:

$$\text{Premature}\;\text{deaths}\;\text{averted}={\text{N}}_{\text{H,T,BL}}\times \left(1-\text{RR}\right)$$

## Data sources

Data from the WHO, the CRASH-2 trial and a systematic review of literature published since 2004 were used to parameterise the equations. The number of trauma deaths for each country, were obtained from the WHO for the year 2008, the most recent year for which data were available. Blunt trauma deaths were estimated by adding the number of deaths from road traffic crashes, falls and other unintentional injuries. Penetrating trauma deaths were estimated by adding the number of deaths from violence and war. Deaths from drowning, poisoning, self-inflicted injuries or burns were not included as these injuries are not usually associated with life-threatening bleeding. Estimates of the proportion of trauma deaths that are in-hospital and the proportion caused by bleeding were based on data from the CRASH-2 trial and from studies identified through a systematic review.

### Systematic review methods

We searched for studies containing original data describing the epidemiology of trauma deaths. We searched MEDLINE, EMBASE and Cab Abstracts on 2 March 2011 using a combination of subject headings and key words based on the following terms; injuries, trauma, mortality, death, fatality, burden, epidemiology. We searched the internet and checked the reference lists of eligible articles. The searches were not restricted by language or publication status. To improve the applicability of the extracted data to the current patterns in trauma death epidemiology, we limited our search to studies published since 2004.

Record screening, full text review and data extraction were performed independently by two authors (KK and JK), with any disagreements resolved through discussion. Data were extracted on study design, setting, sample size, the proportions of deaths occurring in hospital and due to bleeding, using a pre-designed form. Studies that did not provide data on any of the parameters of interest were excluded.

To obtain a summary estimate for each parameter, the study proportions were transformed according to the Freeman Tukey variant of the arcsine square root transformed proportions to correct for over-dispersion [10]. Pooled proportions were calculated as the back-transformation of the weighted mean of the transformed proportions using the random effects model [11].

### Data analysis

For each country, the number of in-hospital trauma deaths due to bleeding was calculated by applying the corresponding estimated proportions to the mortality data, as described above. For the primary analysis, the relative risk reduction from the CRASH-2 trial was applied to estimate the number of premature deaths that could be averted (1) if all patients received TXA within one hour of injury, and (2) if all patients received TXA within three hours of injury. The numbers of deaths averted in each country were combined to give an overall global estimate. To identify the countries with the greatest potential for benefit from TXA, countries were ranked in order of the estimated number of premature deaths averted.

To investigate the impact on the results of uncertainty in the parameter estimates used in the modelling, a number of sensitivity analyses were conducted. First, the analysis was repeated using the lower and upper bounds of the 95% confidence intervals for the parameter estimates to explore the effect of parameter uncertainty. Second, we repeated the analysis using the relative risk estimate for all-cause mortality rather than death due to bleeding. This analysis was conducted to take account of the possibility that some patients who do not die from bleeding because of TXA administration would nevertheless die of other causes such multi-organ failure or brain injury. Third, we repeated the analyses using the relative risk estimate for all-cause mortality with TXA when given at any time within eight hours of injury. Although, previously published subgroup analyses show that early treatment is more effective it is possible that treatment within three hours is not possible in some settings.

For each estimate, to reflect statistical uncertainty around the relative risks of TXA, an uncertainty range was estimated by calculating the numbers of deaths averted based on the 95% confidence intervals for the relative risks. The analyses were conducted using Microsoft Excel and STATA 11 (TX: StataCorp LP) software.

## Results

### Estimation of the effect of TXA on death due to bleeding by geographical region

**Figure 1:**
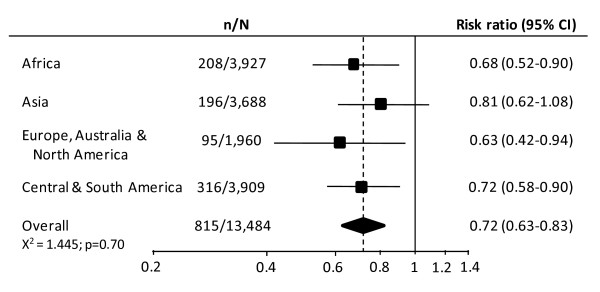
**Risk ratio (95% CI) for death due to bleeding with TXA given within three hours of injury, overall and by geographical region**.

Figure 1 shows the effect of TXA given within three hours of injury on death due to bleeding by geographical region. There was no evidence for heterogeneity in the effect of TXA by region (χ^2 ^= 1.445; *p *= 0.70). The overall RRs for the effect of TXA on death due to bleeding when given within one hour (RR = 0.68; 95% CI 0.57 to 0.82) and within three hours (RR = 0.72; 95% CI 0.63 to 0.83) of injury were therefore taken as the most reliable guide as to the approximate RRs in all regions, and were used to estimate the number of deaths that could be averted with TXA.

### Estimation of the annual number of in-hospital trauma deaths due to bleeding

**Figure 2:**
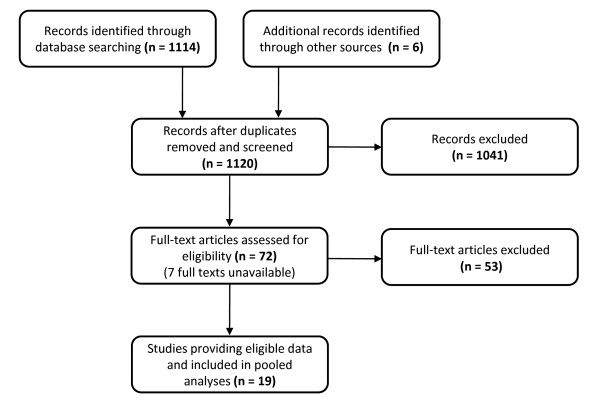
**Flow diagram of the study selection process for systematic review**.

We identified 18 studies, described in 17 reports [12-28], which presented data on the parameters of interest and were included in the systematic review. Studies were conducted in 13 countries; USA, Canada, UK, Australia, Brazil, Denmark, Norway, Mozambique, South Africa, Italy, France, Spain and India. In addition, we obtained data collected as part of the CRASH-2 trial, which recruited patients from hospitals in 40 countries throughout the world. The study selection process is summarised in Figure 2. Data extracted from the studies are summarised in Additional File 1.

Fourteen studies [13-15,17,19-27] involving 24,831 trauma deaths provided data on the proportion of deaths occurring in-hospital; the pooled proportion was 44% (95% CI 33 to 56%). Five studies [3,12,16,18,28] involving 9684 deaths presented data on the proportion of blunt trauma deaths due to haemorrhage; the pooled proportion was 18% (95% CI 13 to 23%). Four studies [3,12,16,28] involving 2256 deaths presented data on the proportion of penetrating trauma deaths due to haemorrhage; the pooled proportion was 55% (95% CI 49 to 62%).

**Figure 3:**
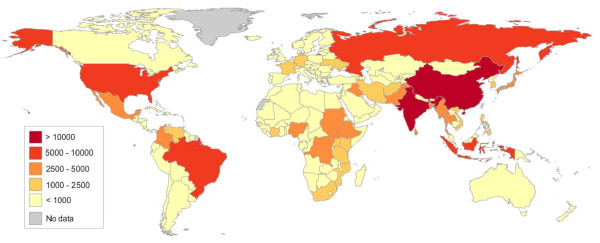
**Global distribution of number of deaths averted with TXA administration within three hours of injury**.

After applying these parameter estimates to the WHO data, we estimate that worldwide every year approximately 400,000 trauma patients die in-hospital from bleeding. If all of these patients receive TXA within one hour of injury the about 128,000 (uncertainty range [UR] ≈ 72,000 to 172,000) deaths could be averted. If all of these patients receive TXA within three hours of injury about 112,000 (UR ≈ 68,000 to 148,000) deaths could be averted. The global distribution of number of premature deaths averted by TXA when administered within three hours of injury is shown in Figure 3.

Results for the countries where more than 1000 deaths could be averted are shown in Table 1. The largest numbers of deaths from haemorrhage and consequently the largest numbers of deaths averted are in Asia. The largest numbers of premature deaths averted are in India (*TXA ≤ 1 hr *≈ 19,000; *TXA ≤ 3 hrs *≈ 16,500) and China (*TXA ≤ 1 hr *≈ 17,000; *TXA ≤ 3 hrs *≈ 15,000). When ranked by the number of premature deaths potentially averted, nine of the top ten countries are low or middle income, the exception being the USA where approximately 4,000 and 3,500 deaths would be averted by TXA given within one hour and three hours of injury, respectively.

**Table 1:** Estimated number of premature trauma deaths averted by TXA per year

	In-hospital trauma deaths from bleeding	Deaths averted TXA ≤ 1 hour	Deaths averted TXA ≤ 3 hours
Worldwide	400,467	128,149	112,131
*Countries with > 1000 deaths averted*			
India	58,801	18,816	16,464
China	54,241	17,357	15,187
Brazil	19,187	6,140	5,372
Russian Federation	16,731	5,354	4,685
Myanmar	13,193	4,222	3,694
Iraq	12,786	4,091	3,580
USA	12,489	3,996	3,497
Indonesia	11,033	3,531	3,089
DR Congo	9,373	2,999	2,624
Sri Lanka	8,979	2,873	2,514
Pakistan	8,770	2,806	2,456
Ethiopia	8,768	2,806	2,455
Nigeria	8,258	2,643	2,312
Colombia	7,348	2,352	2,058
Sudan	7,292	2,334	2,042
Bangladesh	7,210	2,307	2,019
Mexico	7,059	2,259	1,976
Philippines	6,119	1,958	1,713
Thailand	5,572	1,783	1,560
Afghanistan	4,774	1,528	1,337
Uganda	4,620	1,478	1,294
South Africa	4,245	1,359	1,189
Venezuela	4,172	1,335	1,168
Kenya	4,029	1,289	1,128
Tanzania	3,969	1,270	1,111
Iran	3,921	1,255	1,098

### Sensitivity analyses

When the analyses were repeated using the values of the lower and upper 95% CIs of the pooled parameter estimates, the global number of deaths averted ranged from approximately 76,000 to 198,000 if TXA is given within one hour of injury and from 67,000 to 173,000 if given with three hours of injury. When the analysis was carried out using the relative risk estimate for all-cause mortality if TXA is given within one hour (RR = 0.87; 0.78 to 0.97) and within three hours (RR = 0.87; 0.80 to 0.94) of injury, the number of premature deaths averted was 52,000 (TXA ≤ 1 hr ≈ 12,000 to 88,000; TXA ≤ 3 hrs ≈ 24,000 to 80,000). When the analysis was repeated using relative risk estimate for death due to bleeding when TXA is given at any time within eight hours of injury (RR = 0.85; 0.76 to 0.96), the number of premature deaths averted was 60,000 (UR ≈ 16,000 to 96,000). Finally, using the relative risk estimate for all-cause mortality when TXA is given within eight hours of injury (RR = 0.91; 0.85 to 0.97), an estimated 36,000 (UR ≈ 12,000 to 60,000) premature deaths could be averted.

## Discussion

Based on WHO mortality data and a systematic review of the literature we estimate that there are about 400,000 in-hospital deaths from bleeding each year worldwide. If all hospitalised bleeding trauma patients could be treated with TXA within an hour of injury then up to 128,000 of these premature deaths could be averted. If they could be treated within three hours of injury then up to 112,000 premature deaths could averted. Although there is considerable uncertainty in the estimates even the most conservative suggest that tens of thousands of deaths could be averted every year.

We found no compelling evidence that the effect of TXA on death due to bleeding varies by geographical region. Our conclusion is based on a statistical test of interaction which is considered to be the most appropriate way to evaluate subgroup effects [29]. As recommended by methodologists, we pre-specified that unless there was strong evidence against the null hypothesis of homogeneity of effects (i.e. *p *< 0.001), that the overall risk ratio (RR) would be considered to be the most reliable guide to the approximate RRs in all regions. We found no statistical basis to reject the null hypothesis.

The data sources used to parameterise the model are subject to a number of limitations which may have affected our results. First, although the WHO database provides the best available country-level mortality data, poor coverage and coding of mortality registration systems may affect the accuracy of the number of trauma deaths for some countries. Second, our classification of trauma deaths into blunt or penetrating trauma based on the cause of death categories in the WHO data was somewhat arbitrary and would have resulted in some misclassification. However, in the absence of accurate country-specific data, we judged that this approach would provide the most reliable estimates. Third, due to the absence of country-specific data for the proportions of deaths occurring in hospital and the proportion of deaths caused by haemorrhage, we chose to apply average global estimates. We were therefore unable to incorporate between-country variations in these parameter estimates into our analysis. Nevertheless, our estimates were derived from a systematic review of the recent literature and data from the CRASH-2 trial, and thus represent the most accurate estimates available. We also performed sensitivity analyses to assess the impact of uncertainty around the parameter estimates.

Since many deaths from self-inflicted injuries are not usually associated with life-threatening haemorrhage (e.g. self-poisoning, hanging) we excluded this category to avoid over-estimating the number of deaths due to bleeding. However, this is likely to have led to the exclusion of some self-inflicted deaths that were associated with haemorrhage, in which case we may have underestimated the potential of TXA administration.

Our analysis was based on a number of assumptions. We have assumed that there was no use of TXA as a treatment for traumatic bleeding prior to publication of the CRASH-2 trial results. It is possible that a small proportion of the trauma deaths in our sample did receive TXA prior to their death, which may over-estimate the number of deaths averted. However, given that any such prior use of TXA would have been minimal it is unlikely to have greatly affected our overall estimates.

The objective of our analysis was to estimate the potential number of deaths that could be averted assuming TXA use under optimal conditions, that is, when administered appropriately and within three hours of injury, to all eligible bleeding trauma patients. It is unrealistic that such conditions are consistently and fully achieved in clinical practice. For example, the opportunity to treat some eligible patients will be missed and errors in the dose used or its administration may reduce the beneficial effect of TXA.

We assumed that the results of the CRASH-2 trial could be extrapolated to all hospitalised bleeding trauma patients. The CRASH-2 trial used clinical criteria to recruit a large number of patients from 274 hospitals in 40 countries, which helps the results to be generalised widely. Whilst we acknowledge that the underlying risk of death will vary in different settings, this does not necessarily imply that that the relative effect will vary. Indeed, relative effects are often remarkably homogeneous despite differences in underlying risk. This is supported by empirical evidence from a range of trials in which the relative effects are constant across variations in baseline risk [30]. Furthermore, there is no reason to suppose that the mechanism of action of TXA would vary in different populations. However, we acknowledge that the appropriateness of such extrapolation is a matter of judgement.

A further assumption is that all trauma patients reached hospital in time to receive early treatment with TXA; that is either within one hour or within three hours of injury. Such a time frame is unlikely to be realistic in many settings where long distances and other logistical difficulties may delay arrival at hospital. For this reason we performed sensitivity analyses based on the relative effect of TXA from a more conservative estimate of time-to-treatment of within eight hours of injury, the results of which still suggest that up to 60,000 deaths could be averted. Besides, there is reason to predict that time between injury and treatment would be shorter in clinical practice than in the CRASH-2 trial as delays caused by consent procedures would be avoided [31].

In applying the RR of death due to bleeding in our primary analysis we assumed that all deaths in this group would be avoided. However, it is possible that whilst TXA may prevent death due to bleeding, some patients would die from other causes instead. If this is the case, then our primary analysis would over-estimate the number of death averted. To address this we performed a sensitivity analysis in which the effect of TXA on all-cause mortality was used. Even using this smaller relative reduction, up to 50,000 deaths could be averted.

We restricted our analysis to the potential benefit of in-hospital use of TXA. However, our parameter estimate of the proportion of in-hospital trauma deaths indicates that most trauma deaths occur before arrival at hospital. TXA is a practicable treatment suitable for use in a range of health-care settings, including pre-hospital. If TXA was used in the pre-hospital setting then many more premature deaths might be averted.

## Conclusions

Our analysis shows the potential of TXA to reduce trauma deaths worldwide. Realisation of this potential is likely to require further efforts in dissemination and implementation, particularly in low and middle income settings.

## Competing interests

The authors declare that they have no competing interests.

## Authors' contributions

KK, JK and IR designed the study. KK, JK and PP obtained the data and conducted all analyses with advice from PE and IR. KK wrote the paper with input from all other authors. All authors had full access to all the data in the study and had final responsibility for the decision to submit for publication. All authors read and approved the final manuscript.

## Pre-publication history

The pre-publication history for this paper can be accessed here:

http://www.biomedcentral.com/1471-227X/12/3/prepub

## Supplementary Material

Additional file 1 **Summary of data extracted from studies included in systematic review**. Click here for file

## References

[B1] WHO Injuries & violence: the facts 2010 Geneva22393171

[B2] Sauaia A Moore FA Moore EE Moser KS Brennan R Read RA Pons PT Epidemiology of trauma deaths: a reassessment J Trauma 1995 38 2 185 193 10.1097/00005373-199502000-00006 7869433

[B3] The CRASH-2 Collaborators Effects of tranexamic acid on death, vascular occlusive events, and blood transfusion in trauma patients with significant haemorrhage (CRASH-2): a randomised, placebo-controlled trial Lancet 2010 376 9734 23 32 20554319 10.1016/S0140-6736(10)60835-5

[B4] The CRASH-2 Collaborators The importance of early treatment with tranexamic acid in bleeding trauma patients: an exploratory analysis of the CRASH-2 randomised controlled trial Lancet 2011 377 9771 1096 1101 1101 e1091-1092 21439633 10.1016/S0140-6736(11)60278-X

[B5] Guerriero C Cairns J Perel P Shakur H Roberts I Cost-effectiveness analysis of administering tranexamic acid to bleeding trauma patients using evidence from the CRASH-2 trial PLoS One 2011 6 5 e18987 10.1371/journal.pone.0018987 21559279 PMC3086904

[B6] WHO Summary of the report of the 18th meeting of the WHO Expert Committee on the Selection and Use of Essential Medicines 2011 Geneva

[B7] British Forces News Injured soldiers in Afghanistan saved by blood-clotting drug 19.01.11 2011 http://www.youtube.com/watch?v=oj6P2cwwRYw

[B8] Peden M McGee K Sharma G The injury chart book: a graphical overview of the global burden of injuries 2002 Geneva: World Health Organization

[B9] Kauvar DS Wade CE The epidemiology and modern management of traumatic hemorrhage: US and international perspectives Crit Care 2005 9 Suppl 5 S1 S9 10.1186/cc3779 16221313 PMC3226117

[B10] Trinquart L Touze E Pitfalls in meta-analysis of observational studies: lessons from a systematic review of the risks of stenting for intracranial atherosclerosis Stroke 2009 40 10 e586 e587 author reply e590 10.1161/STROKEAHA.109.556290 19745174

[B11] DerSimonian R Laird N Meta-analysis in clinical trials Control Clin Trials 1986 7 3 177 188 10.1016/0197-2456(86)90046-2 3802833

[B12] Boulanger L Joshi AV Tortella BJ Menzin J Caloyeras JP Russell MW Excess mortality, length of stay, and costs associated with serious hemorrhage among trauma patients: findings from the National Trauma Data Bank Am Surg 2007 73 12 1269 1274 18186388

[B13] Demetriades D Kimbrell B Salim A Velmahos G Rhee P Preston C Gruzinski G Chan L Trauma deaths in a mature urban trauma system: is "trimodal" distribution a valid concept? J Am Coll Surg 2005 201 3 343 348 10.1016/j.jamcollsurg.2005.05.003 16125066

[B14] Demetriades D Murray J Charalambides K Alo K Velmahos G Rhee P Chan L Trauma fatalities: time and location of hospital deaths J Am Coll Surg 2004 198 1 20 26 10.1016/j.jamcollsurg.2003.09.003 14698307

[B15] Di Bartolomeo S Sanson G Michelutto V Nardi G Burba I Francescutti C Lattuada L Scian F Epidemiology of major injury in the population of Friuli Venezia Giulia-Italy Injury 2004 35 4 391 400 10.1016/S0020-1383(03)00246-8 15037374

[B16] Dutton RP Stansbury LG Leone S Kramer E Hess JR Scalea TM Trauma mortality in mature trauma systems: are we doing better? An analysis of trauma mortality patterns, 1997-2008 J Trauma 2010 69 3 620 626 10.1097/TA.0b013e3181bbfe2a 20093983

[B17] Evans JA van Wessem KJ McDougall D Lee KA Lyons T Balogh ZJ Epidemiology of traumatic deaths: comprehensive population-based assessment World J Surg 2010 34 1 158 163 10.1007/s00268-009-0266-1 19882185

[B18] Gilroy D Deaths from blunt trauma, after arrival at hospital: plus ca change, plus c'est la meme chose Injury 2005 36 1 47 50 10.1016/j.injury.2004.04.015 15589912

[B19] Gomez D Berube M Xiong W Ahmed N Haas B Schuurman N Nathens AB Identifying targets for potential interventions to reduce rural trauma deaths: a population-based analysis J Trauma 2010 69 3 633 639 10.1097/TA.0b013e3181b8ef81 20016384

[B20] Gomez de Segura Nieva JL Boncompte MM Sucunza AE Louis CL Segui-Gomez M Otano TB Comparison of mortality due to severe multiple trauma in two comprehensive models of emergency care: Atlantic Pyrenees (France) and Navarra (Spain) J Emerg Med 2009 37 2 189 200 10.1016/j.jemermed.2007.10.089 18829202

[B21] Masella CA Pinho VF Costa Passos AD Spencer Netto FA Rizoli S Scarpelini S Temporal distribution of trauma deaths: quality of trauma care in a developing country J Trauma 2008 65 3 653 658 10.1097/TA.0b013e3181802077 18784580

[B22] Meel BL Pre-hospital and hospital traumatic deaths in the former homeland of Transkei, South Africa J Clin Forensic Med 2004 11 1 6 11 10.1016/j.jcfm.2003.10.009 15261006

[B23] Meisler R Thomsen AB Abildstrom H Guldstad N Borge P Rasmussen SW Rasmussen LS Triage and mortality in 2875 consecutive trauma patients Acta Anaesthesiol Scand 2010 54 2 218 223 10.1111/j.1399-6576.2009.02075.x 19817720

[B24] Nizamo H Meyrowitsch DW Zacarias E Konradsen F Mortality due to injuries in Maputo City, Mozambique Int J Inj Contr Saf Promot 2006 13 1 1 6 10.1080/17457300500151705 16537218

[B25] Potenza BM Hoyt DB Coimbra R Fortlage D Holbrook T Hollingsworth-Fridlund P The epidemiology of serious and fatal injury in San Diego County over an 11-year period J Trauma 2004 56 1 68 75 10.1097/01.TA.0000101490.32972.9F 14749568

[B26] Singh B Palimar V Arun M Mohanty MK Profile of trauma related mortality at Manipal Kathmandu Univ Med J (KUMJ) 2008 6 23 393 297 10.3126/kumj.v6i3.1722 20071828

[B27] Soreide K Kruger AJ Vardal AL Ellingsen CL Soreide E Lossius HM Epidemiology and contemporary patterns of trauma deaths: changing place, similar pace, older face World J Surg 2007 31 11 2092 2103 10.1007/s00268-007-9226-9 17899256

[B28] Tien HC Spencer F Tremblay LN Rizoli SB Brenneman FD Preventable deaths from hemorrhage at a level I Canadian trauma center J Trauma 2007 62 1 142 146 10.1097/01.ta.0000251558.38388.47 17215745

[B29] Rothwell PM Treating individuals 2. Subgroup analysis in randomised controlled trials: importance, indications, and interpretation Lancet 2005 365 9454 176 186 10.1016/S0140-6736(05)17709-5 15639301

[B30] Furukawa TA Guyatt GH Griffith LE Can we individualize the 'number needed to treat'? An empirical study of summary effect measures in meta-analyses Int J Epidemiol 2002 31 1 72 76 10.1093/ije/31.1.72 11914297

[B31] Roberts I Prieto-Merino D Shakur H Chalmers I Nicholl J Effect of consent rituals on mortality in emergency care research Lancet 2011 377 9771 1071 1072 10.1016/S0140-6736(11)60317-6 21439634

