# Supplementary material for: Avoidable mortality from giving tranexamic acid to bleeding trauma patients: an estimation based on WHO mortality data, a systematic literature review and data from the CRASH-2 trial
Source: BMC Emerg Med. 2012 Mar 1;12:3. doi: 10.1186/1471-227X-12-3 (PMC3314558; doi:10.1186/1471-227X-12-3)
Supplement: Additional file 1 — Summary of data extracted from studies included in systematic review. [file 1471-227X-12-3-S1.DOC]

| **Author, year** | **Country** | **Design** | **Deaths (n)** | **Deaths occurring in-hospital (n)** | **Blunt trauma deaths due to haemorrhage (n)** | **Penetrating trauma deaths due to haemorrhage (n)** |
| --- | --- | --- | --- | --- | --- | --- |
| Boulanger 2007 | USA | Trauma-registry based study; 2000-2004 | 7362 | - | 19.6 | 56.3 |
| CRASH-2 2010 | Worldwide | Randomised controlled trial; 2005-2010 | 1618* | - | 28.2 | 59.6 |
| Demetriades 2004 | USA | Trauma-registry based study; 1993-2002 | 2648 | 33.4 | - | - |
| Demetriades 2005 | USA | Trauma registry and emergency medical services records based study; Jan 2000-Dec 2002 | 4151 | 79.6 | - | - |
| Di Barolomeo 2004 | Italy | Prospective population-based study; March 1998-Feb 1999 | 286 | 37.8 | - | - |
| Dutton 2010 | USA | Trauma-registry based study; July 1996-June 2008 | 2327 | - | 18.5 | 46.6 |
| Evans 2010 | Australia | Prospective study of autopsies reports and medical records; Feb 2005-Jan 2006 | 175 | 61.1 | - | - |
| Gilroy 2005 | UK | Retrospective study of in-hospital deaths; 2001 | 94** | - | 13.8 | - |
| Gomez de Segura Nieva 2009a | Spain | Prospective study of severe multiple injury patients; April 2001-March 2002 | 165 | 27.3 | - | - |
| Gomez de Segura Nieva 2009b | France | Prospective study of severe multiple injury patients; April 2001-March 2002 | 151 | 33.8 | - | - |
| Gomez 2010 | Canada | Retrospective population-based study; 2002-2003 | 3486 | 46.2 | - | - |
| Masella 2008 | Brazil | Retrospective population based study; Jan 2000 – Dec 2001 | 787 | 43.1 | - | - |
| Meel 2004 | South Africa | Retrospective study of medico-legal autopsies; 1997-1998 | 274 | 25.9 | - | - |
| Meisler 2010 | Denmark | Prospective population based study; 2006 | 2068 | 41.7 | - | - |
| Nizamo 2006 | Mozambique | Respective review of registered deaths; 2000 | 1135 | 38.8 | - | - |
| Potenza 2004 | USA | Retrospective population based study; 1987-1997 | 14,767 | 27.9 | - | - |
| Singh 2008 | India | Retrospective study of autopsy reports; Jan 2001-Dec 2003 | 344 | 75.8 | - | - |
| Soreide 2007 | Norway | Retrospective review of autopsy reports; 1996-2004 | 260 | 48.1 | - | - |
| Tien 2007 | Canada | Retrospective study of in-hospital deaths; 1999-2003 | 558 | - | 8.5 | 61.6 |
